# Exploring the Experiences of Persons Living With Dementia and Their Care Partners in a Memory Café-Style Program

**DOI:** 10.1093/geroni/igaa057.874

**Published:** 2020-12-16

**Authors:** Morgan Bunting, Inga Antonsdottir, Marti Bailey, Quincy Samus

**Affiliations:** 1 Johns Hopkins University, Halethorpe, Maryland, United States; 2 Johns Hopkins University, Baltimore, Maryland, United States; 3 Club Memory, Washington, D.C., United States

## Abstract

Community-based supportive care interventions are needed to address unmet needs and maximize quality of life for persons living with dementias and their care partners (care dyad). Memory cafés and similar programs serve as a potentially important vehicle to reduce dementia stigmatization, improve inclusiveness, promote well-being, socialization and meaningful activity engagement, and disseminate dementia-related education and knowledge. Despite gaining popularity, little is known about how these programs impact participants or how they work best. The Club Memory® program in Washington, D.C. is an enhanced memory café-style program that delivers a normalizing environment, decreased negative stigma and a chance to experience a catalyst for change despite disease progression. Using qualitative methods, we investigated the perceived impact, benefits, and weaknesses of Club Memory from multi-stakeholder perspective, to explore how this program might be improved and standardized for broader implementation. We conducted two focus groups, one of care partners and one of persons living with dementia (N=12). Upon analyzing transcriptions, five common themes emerged among the focus groups: Atmosphere, Sense of Community, Skill-Building, Tools and Strength in Staff. Integrating perspectives from the care dyad revealed congruent thoughts on program perception, benefits and strengths, and opportunities for improvement. Club Memory® appears to be a valuable model that creates a stigma-free supportive environment, educates and engages the care dyad to foster understanding, confidence, curiosity and empowerment. Our findings contribute to a deeper understanding of the impact community-based strategies can positively impact the care dyad’s life.

